# Examining Physical Therapy Students' Subjective Mastery of Simulated Clinical Practice Using Smart Glasses to Share Teacher's Visual Field Information

**DOI:** 10.14789/ejmj.JMJ24-0033-OA

**Published:** 2025-03-12

**Authors:** YOKO TAKAHASHI, TOMOFUMI YAMAGUCHI, YOJI SUMISE, YUJI FUJINO, KENZO MUROI, TETSUYA TAKAHASHI, TOSHIYUKI FUJIWARA, HIROYUKI DAIDA

**Affiliations:** 1 Department of Physical Therapy, Faculty of Health Science, Juntendo University, Tokyo, Japan; 1 Department of Physical Therapy, Faculty of Health Science, Juntendo University, Tokyo, Japan; 2 Department of Faculty of Health Science, Juntendo University, Tokyo, Japan; 2 Department of Faculty of Health Science, Juntendo University, Tokyo, Japan; 3 Department of Radiological Technology, Faculty of Health Science, Juntendo University, Tokyo, Japan; 3 Department of Radiological Technology, Faculty of Health Science, Juntendo University, Tokyo, Japan; 4 Department of Rehabilitation Medicine, Juntendo University Graduate School of Medicine, Tokyo, Japan; 4 Department of Rehabilitation Medicine, Juntendo University Graduate School of Medicine, Tokyo, Japan; 5 Juntendo University, Tokyo, Japan; 5 Juntendo University, Tokyo, Japan; 6 Department of Cardiology, Juntendo University Graduate School of Medicine, Tokyo, Japan; 6 Department of Cardiology, Juntendo University Graduate School of Medicine, Tokyo, Japan

**Keywords:** physical therapy, electrical stimulation, education, smart glasses, electrophysiological agents

## Abstract

**Objectives:**

Physical therapy education depends on hands-on training. However, the COVID-19 pandemic limited face-to-face demonstrations. This study considers the use of smart glasses, devices that allow real-time information sharing from remote locations. We investigated the efficacy of teachers’ use of smart glasses during a class on students’ subjective mastery of applying neuromuscular electrical stimulation (NMES).

**Methods:**

119 second-grade students from the physical therapy department were randomly divided into three groups: smart glasses demonstration combined with video watching (smart glasses, *n* = 40), face-to-face demonstration combined with video watching (face-to-face, *n* = 40), and video watching only (video only, *n* = 39). All groups watched a 10-minute video on NMES guidance. The smart glasses group practiced NMES while viewing a shared demonstration via smart glasses worn by the teacher. The face-to-face group received a demonstration from the teacher before practicing NMES. All groups completed a questionnaire on their mastery of NMES.

**Results:**

In terms of NMES mastery, 85% of students in the smart glasses group scored the highest, followed by 82% in the face-to-face group and 64% in the video only group. The smart glasses group found it significantly easier to view the device’s operating screen during the demonstration than the face-to-face group. Conversely, concentration, and ease of asking questions were significantly higher in the face-to-face group than the smart glasses group.

**Conclusions:**

NMES education using smart glasses could be as effective as face-to-face demonstrations in promoting students’ subjective mastery, but further actions are needed to compensate for the shortcomings.

## Introduction

The operation of physical therapy devices and their application to patients represent important skills in physical therapy. In current physical therapy education, the main method employed by teachers is demonstrating how to operate equipment; subsequently students practice their instructions. In recent years, smart glasses, which are devices that can share visual field information, have been utilized in medical education. When wearing glasses embedded with cameras, real-time visual field information can be displayed on a monitor and remotely shared via the Internet. These glasses are used for training surgical skills^1, 2)^, decision-making in triage^3-5)^, remote medical education of a virtual demonstration of normal vaginal delivery^6)^, and radiography education^7)^. However, they have not been used in physical therapy education. Instead, physical therapy education has relied on face-to-face practical training to operate physical therapy equipment and teach manual techniques. During the coronavirus disease 2019 (COVID-19) pandemic, face-to-face demonstrations were limited because crowding was avoided. Therefore, this study explores how the use of smart glasses may help educate physical therapy students on small physical therapy devices.

Since the COVID-19 pandemic, numerous remote medical education initiatives have been reported^8-24)^. Contrastingly, although many reports on lectures exist, only a few discuss practical training in surgical techniques for students and residents^8, 9)^ and deal with patient examination techniques^10)^; this indicates a lack of remote practical education^11)^.

Furthermore, to our knowledge, there have been no reports of remote learning in physical therapy education. Although the present study consists of simulated remote education, it is significant in verifying the technology that can be utilized for remote education of practical technique.

Accordingly, this study tested the feasibility of using smart glasses during a physical therapy class on neuromuscular electrical stimulation (NMES). This study investigated the students’ subjective mastery degree of NMES through teachers’ use of smart glasses during physical therapy education.

## Materials and Methods

**Figure 1:**
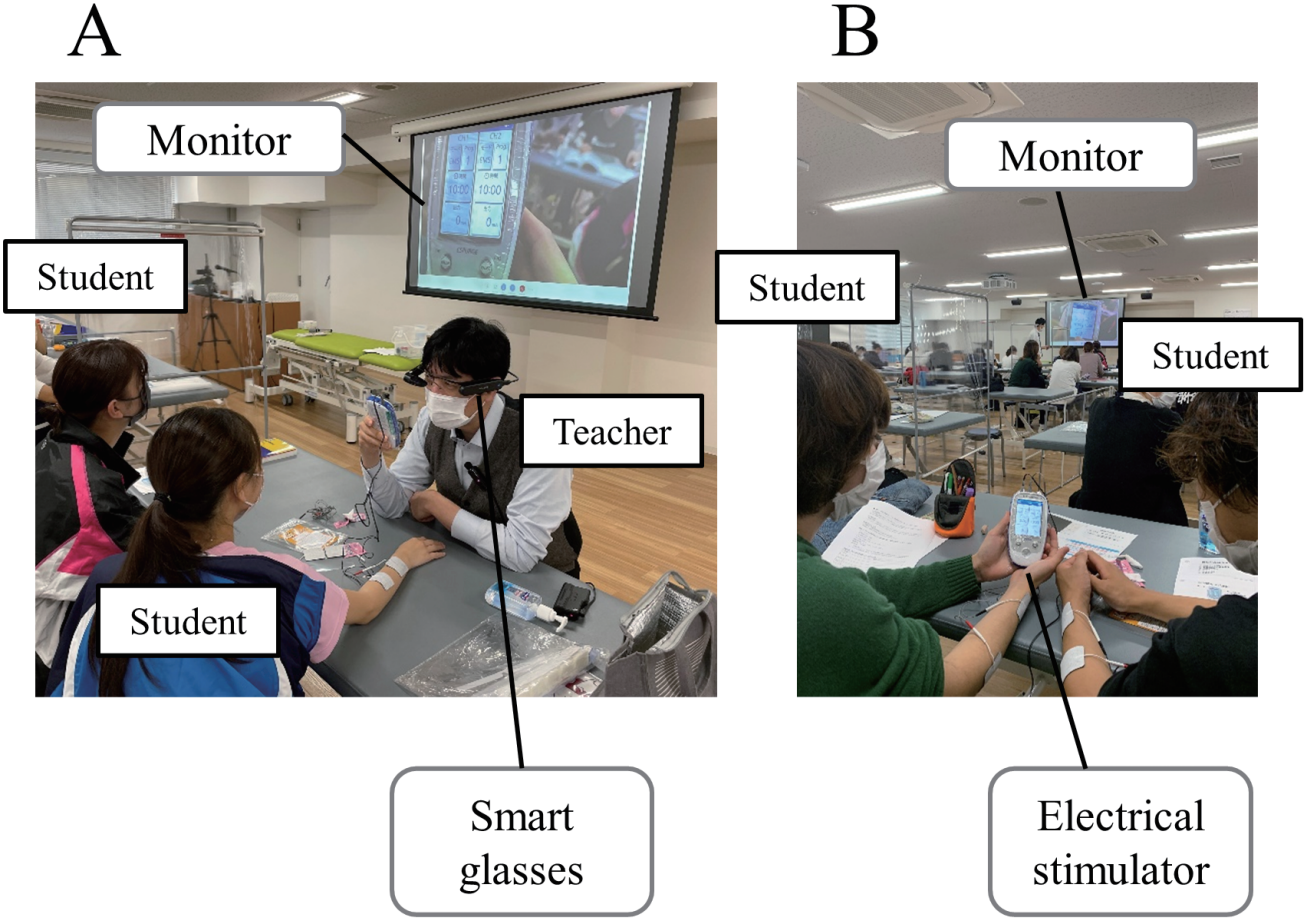
Demonstration using smart glasses A. The teacher put on smart glasses and projected his field of view on a large screen monitor as he operated the device and attached electrodes to a student's forearm. B. The 40 students practiced operating the stimulator and applying it to another student while watching the teacher's field of view on a large screen monitor. Three large screen monitors were set up for every 40 students, who could see the monitors from their seats.

The study design was approved by the local ethical review committee at Faculty of Health Science, Juntendo University (Approval number: 22-033) and was carried out in accordance with the Declaration of Helsinki. Participants’ informed consent regarding the use of class content was obtained before the data analysis. This study also obtained written permission to use a photograph for this study from the teacher and students depicted therein (Figure 1). The study was retrospectively analyzed after conducting the class.

**Figure 2:**
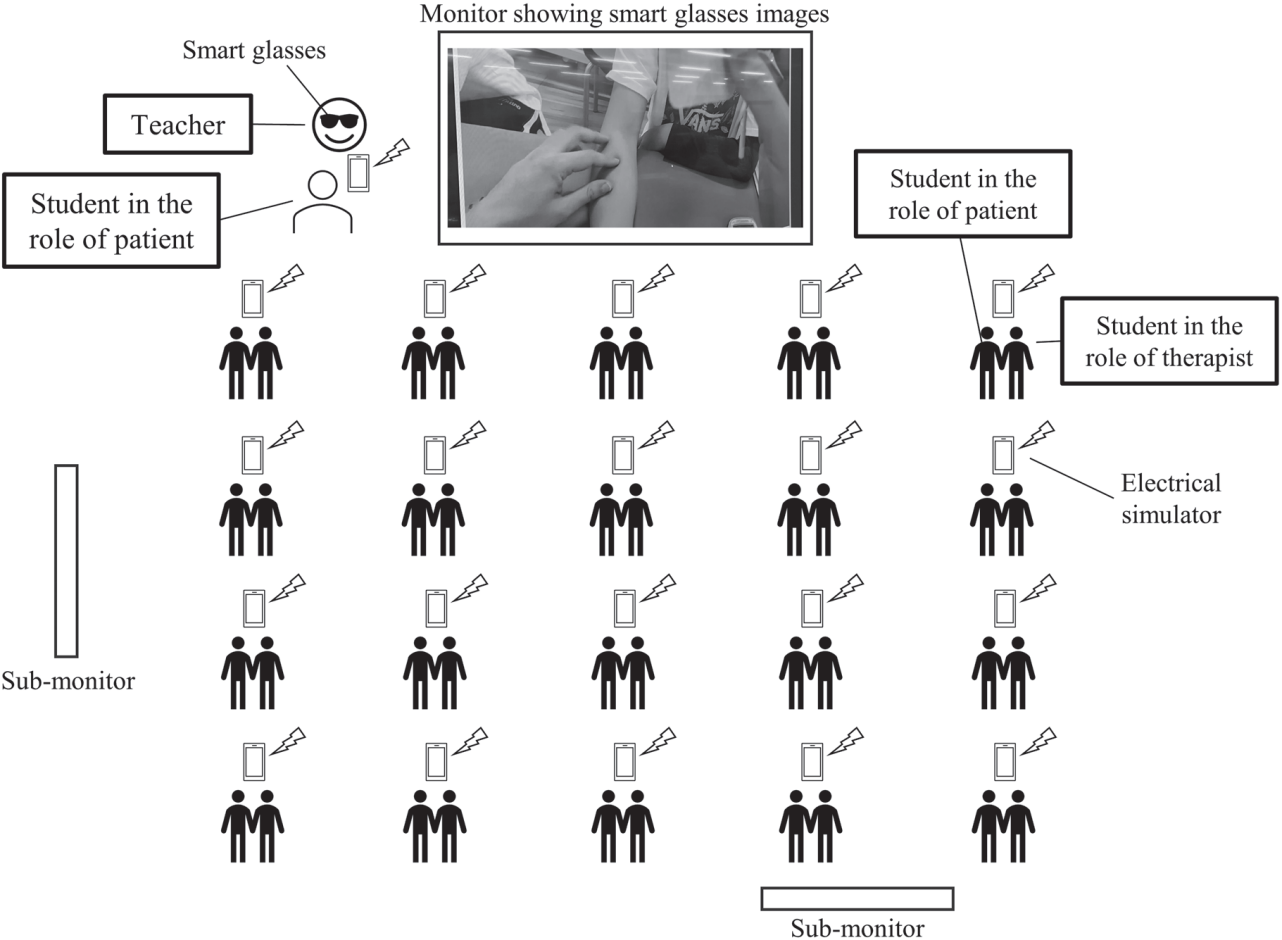
Shema of demonstration in the smart glasses group The 40 students practiced operating the stimulator and applying it to their partner while watching the teacher's field of view on the monitor. To ensure that all students had a good view of the monitors, the same images were played on two sub-monitors in addition to the main monitor. Students were paired as a patient and a therapist and used one electrical stimulator.

The sample included 119 second-grade students from Physical Therapy Department, Faculty of Health Science, Juntendo University. The study design was a parallel group randomized controlled trial. This study was conducted as part of the “Practice of Electrophysical Agents” curriculum. The objectives of this subject are: 1. to understand the biological reactions, indications, and contraindications to electrophysiological agents and to be able to select the appropriate electrophysiological agents for the patient’s condition; 2. to operate the equipment used in electrophysiological agents according to the appropriate procedures; and 3. to explain logically the effects of electrophysiological agents based on the biological reactions before and after the treatment. The class conducted present study focused on physical therapy using a small electrical simulator, ESPURGE (ITO ESPURGE, Ito Co., Ltd, Saitama, Japan), and its human application. All students were randomly divided into three groups: smart glasses demonstration combined with video watching (smart glasses group, *n* = 40), face-to-face demonstration combined with video watching (face-to-face group, *n* = 40), and video watching only (video only group, *n* = 39). Block randomization was used for grouping. Students were randomly assigned to 3 blocks of up to 40 people using the RAND function in Microsoft Excel (Microsoft Corporation, Redmond, WA, USA). The grouping process was conducted by a teacher who was primarily responsible for NMES practice. One device was distributed per two students, who simulated the roles of physical therapist and patient. At the beginning of the class, all groups watched a 10-minute video on operating the electrical stimulator. The video included the following: 1) purpose of NMES practice, 2) function of the device, 3) how to set up the device, 4) how to apply the device to the patient, and 5) precautions for NMES operation. In addition to text and video, video narration by a teacher was included. The smart glasses and face-to-face groups watched demonstrations on operating the device, applying electrodes on the skin, and palpating the muscles that accompanied the electrode application. The teacher in charge of the demonstration had 5 years of education experience in the Practice of Electrophysical Agents and 17 years of experience as a physical therapist. The smart glasses group simultaneously practiced the use of the electrical stimulator and watched a demonstration in which the teacher’s visual field was shared via smart glasses. The teacher wore VUSIX M400 smart glasses and projected his field of view to a large screen monitor as he operated the device and attached electrodes to the student in the role of patient (Figure 1). The 40 students practiced operating the stimulator and applying it to their partner while watching the teacher’s field of view on the monitor. The practice of the smart glasses group is shown in Figure 2. To ensure that all students had a good view of the monitors, the same images were played on two sub-monitors in addition to the main monitor. In the face-to-face group, 40 students gathered around the teacher to watch the demonstration and then practiced using the electrical stimulator. During the individual practice sessions after the video watching or demonstrations, the teacher walked among the students and helped them practice.

At the end of the class, all students answered a questionnaire (Table 1). This questionnaire was developed through discussions among the teachers in charge of the classes. Prior to the class, the teachers conducted a pre-test of the practical training using smart glasses in the classroom where the practical training was to be conducted. Based on this experience, we listed the options for the smart glasses demonstration, referring to their impressions, compared to those for the face-to-face demonstration. In addition, the questionnaire was prepared with reference to previous studies on students’ impressions in e-learning^25)^. The acquisition and analysis of the questionnaires were conducted by a different teacher than the one who conducted the class demonstrations and groupings. The questionnaire was self-administered electronically using Google forms. All groups were asked about their subjective mastery degree of NMES on a scale from 1 to 5 (Q1). Score 5 means ‘I learned well.’ The smart glasses and face-to-face groups were asked two questions: whether watching the video alone was sufficient for their understanding and whether their understanding was enhanced by both watching the video and attending the demonstration (Q2). Students who chose the latter option were asked to explain their reasoning using multiple options (Q3). The following options were established to clarify the benefits of smart glasses and face-to-face demonstration: 1) The technique was easy to see. 2) I felt a sense of realism and maintained my concentration. 3) The operation screen of the device was easy to see. 4) It was easy to ask questions if there was something I did not understand while operating the device. 5) Operating procedures were easy to understand because I was able to operate the device at the same time as the teacher (the smart glasses group only). In Q3, students were able to choose multiple options. Further, the smart glasses group was asked whether the demonstration using smart glasses had helped them learn about NMES. There was a section for free opinions at the end of the questionnaire.

A χ^2^ test was performed to test for gender bias in each group. The percentages of students in the smart glasses and face-to-face groups who chose each option were compared using the Mann-Whitney U test. IBM SPSS statistics 21 was used for statistical analysis.

**Table 1:** Contents and results of the questionnaire

Questions		Video-watching only group, %	Face-to-face group, %	Smart glasses group, %
Q1. Please rate your mastery degree of NMES from 1-5 (1 = I did not learn at all, 5 = I learned well)		5: 64 4: 28 3: 8	5: 82 4: 15 3: 3	5: 85 4: 15
Q2. After learning about NMES, did you understand it well enough, even if you only watched a video, or did you understand it well enough from the video and demonstration?	Options			
	Even if it was just the video, I think I understood enough.	N/A	5	5
	I understood it well after both the video watching and demonstration.	N/A	95	95
Q3. Please only answer if you selected “I understood it well after both the video watching and demonstration” in Q2. Why did you choose this option? (You can select multiple reasons) (*n* = 38 in each group)	Options			
	The technique was easy to see.	N/A	76	73
	I felt a sense of realism and maintained my concentration.	N/A	53	18
	The operation screen of the device was easy to see.	N/A	50	87
	It was easy to ask questions if there was something I did not understand while operating the device.	N/A	53	0.1
	(Smart glasses group only) Operating procedures were easy to understand because I was able to operate the device at the same time as the teacher.	N/A	N/A	95
(Smart glasses group only) Did the demonstration using smart glasses help you to understand NMES?		N/A	N/A	Yes: 100

Abbreviations: NMES, neuromuscular electrical stimulation; N/A, not available.

## Results

**Figure 3:**
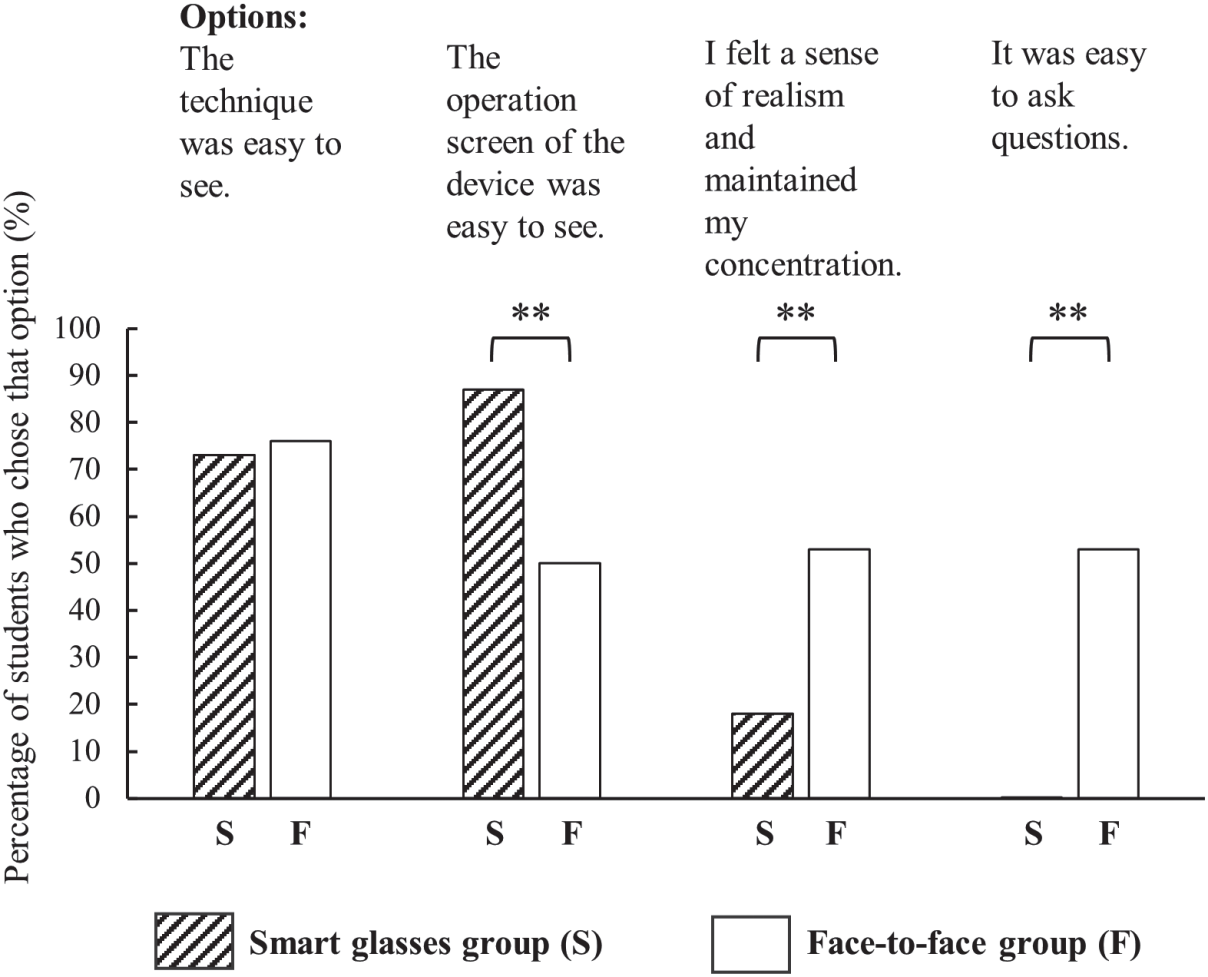
A statistical comparison between the face-to face and smart glasses groups in terms of the percentage of reasons chosen for the following questions: ‘please only answer if you selected “I understood it well after the video watching and demonstration” in Q2. Why did you choose this option? (You can select multiple reasons)’ Students in smart glasses demonstration combined with video watching (smart glasses group) and face-to-face demonstration combined with video watching (face-to-face group) who indicated that they liked that there was a demonstration as well as a video were asked why. The four options were as follows: 1) The technique was easy to see, 2) The operation screen of the device was easy to see, 3) I felt a sense of realism and maintained my concentration, and 4) It was easy to ask question if there was something I did not understand while operating the device. The shaded bars indicate the smart glasses group and the white bars indicate the face-to-face group. ** indicates p < .01 with Mann-Whitney U test. The percentage of those who responded “The operation screen of the device was easy to see” was significantly higher (p = .004) in the smart glasses group (87%) compared to the face-to-face group (50%). Conversely, the percentages of respondents who chose the reasons “I felt a sense of realism and maintained my concentration.” (p = .004) and “it was easy to ask questions if there was something I did not understand while operating the device.” (p < .001) were significantly higher in the face-to-face group than in the smart glasses group.

Questionnaire answers of all 119 students in the class were valid. The age range of the students in the study was 19-20 years. The gender ratio (male: female) for each group was 0.38: 0.62 for the smart glasses group, 0.48: 0.52 for the face-to-face group, and 0.49: 0.51 for the video only group. The χ^2^ test showed no significant difference in the gender ratio between the groups (p = .543). Table 1 shows that, for the mastery degree of NMES, 85% of the smart glasses group answered “5: I learned well,” followed by 82% in the face-to-face group and 64% in the video only group. Some respondents in the face-to-face and video only groups gave a score of 3 (= normal), but only 4 (= I learned a little) and 5 (= I learned well) in the smart glasses group. A statistical comparison between the face-to-face and smart glasses groups in terms of the percentage of reasons chosen for the following questions is shown in Figure 3: ‘please only answer if you selected “I understood it well after the video watching and demonstration” in Q2. Why did you choose this option? (You can select multiple reasons)’. In both the smart glasses and face-to-face groups, 95% responded “I understood it well after both the video watching and demonstration.” The percentage of those who responded “The operation screen of the device was easy to see” was significantly higher (p = .004) in the smart glasses group (87%) compared to the face-to-face group (50%). Conversely, the percentages of respondents who chose the reasons “I felt a sense of realism and maintained my concentration.” (p = .004) and “it was easy to ask questions if there was something I did not understand while operating the device.” (p < .001) were significantly higher in the face-to-face group than in the smart glasses group. Moreover, 95% of the smart glasses group answered “Operating procedures were easy to understand because I was able to operate the device at the same time as the teacher.” All students in the smart glasses group stated that the smart glasses demonstration helped them with their understanding of NMES. In the free opinions section, it was pointed out that delays in video transmission occasionally occur in the smart glasses group.

## Discussion

The results revealed that teaching students about NMES via smart glasses enhanced their subjective mastery as much as face-to-face demonstrations. This study is the first report of the use of smart glasses for simulated clinical practice in physical therapy.

In the two groups attending the demonstration (smart glasses and face-to-face), most students stated that the combination of video watching and demonstration helped them learn more compared to exclusively watching the video. The smart glasses group mentioned that this was due to the clear visibility of the device’s operation screen and the ability to operate the device simultaneously with the teacher. Considering that the device had a small operation screen, it may have been difficult for 40 students to simultaneously view the screen. Thus, the teachers’ use of the smart glasses could have helped the students to see the controls, as they could see the teachers’ hands operating the device projected on a large screen. While video cameras could have been used to capture the teachers’ hands, the smart glasses may have made it easier to switch between looking at the device and at the teachers’ hands. A previous study using smart glasses for surgeon skill transfer reported that although they were able to support general surgical decision making, the reduced resolution of the images and the delay in video transmission made real-time guidance by a remote instructor in line with anatomical landmarks difficult^2)^. In the present study, the resolution of the smart glasses was considered sufficient for recognition of the device and procedures captured. However, depending on the content of practical training, the choice of smart glasses may not be suitable. In situations where high resolution is required, measures such as the combination of still image capture are required^2)^. One of the features of smart glasses is that watchers can experience the wearer’s field of view as if it were their own. In the fields of rehabilitation and neuroscience, virtual reality has been used in some cases as a way to enhance a sense of agency^26)^. While not as immersive as virtual reality, smart glasses could provide a greater sense of agency than face-to-face demonstrations in that they can experience the wearer’s field of view. Unlike virtual reality, smart glasses are easy because only educators need to wear the device. As there is no research to our knowledge on the use of smart glasses in medical education and their relationship with sense of agency, future verification is needed.

The students in the smart glasses group pointed out that delays in video transmission occasionally occur because of network error. As a disadvantage of the hardware aspect of the smart glasses, there was a delay in video transmission due to a delay in Wi-Fi connection. Muroi et al^7)^ also stated that network errors during the use of smart glasses. This can also happen if another device is connected to the same Wi-Fi network at the same time. In a student satisfaction survey of remote learning, internet delay was also reported to decrease student satisfaction^25)^. When conducting clinical education remotely, it is necessary to constantly monitor video transmission delays to students at a distance and to ensure a stable network.

Meanwhile, the face-to-face demonstration was more significantly helpful in maintaining students’ concentration and enhancing the extent to which they were at ease with asking questions. Additionally, these factors could have been attributed to the physical closeness between the teacher and students. These problems may also occur in the future when remote clinical education using smart glasses is conducted. In previous studies, it has been reported that difficulty in asking the teacher questions decreases satisfaction with remote learning^25, 27)^. Therefore, when conducting remote practice, it is necessary to provide frequent opportunities for students to ask questions to ensure that students are comfortable asking questions. In addition, interactive communication and practice between teachers and students may be a good way to keep students focused with smart glasses. Overall, face-to-face demonstrations and smart glasses utilization have advantages and disadvantages, therefore, they should be used accordingly. For example, in contexts where people want to avoid crowds, such as during a pandemic, a demonstration using smart glasses could be a suitable alternative to a face-to-face class.

In this study, the percentage of students from the video only group who scored the highest in subjective mastery was lower than for the other two groups, but no students scored 1 or 2. Since the video explained everything from how to operate the device to its application to patients, basic understanding was obtained, but subsequent skill retention may have been poorer in the video only group than in the other two groups. In some aspects, e-learning through video viewing is viewed favorably by students because the videos can be replayed repeatedly and can be viewed at their pace^28)^. Thus, using videos for preparation and review may enhance skill acquisition.

Since the COVID-19 pandemic, numerous remote medical education initiatives have been reported^8-24)^. However, these reports relate to learning, and research on remote education in clinical practice has not progressed. The few reports of clinical practice education^8-10)^ utilize remote systems such as zoom. In this study, the simulation of clinical education was carried out in the on-campus practice, and as the next step, it will be developed into the clinical education connecting the clinical practice and the university by the smart glasses. Furthermore, both students^29)^ and teachers^30)^ feel that although a certain level of understanding of manual skills, such as manual therapy, can be achieved through video and remote learning, the lack of peer practice and immediate faculty support limits mastery. In an investigation conducted in manual therapy practice, it was reported that learning from the instructor and practicing with fellow students was superior to learning from a video and practicing with a dummy in terms of learning to perform manual therapy^29)^. How to solve the problem of manual skill mastery in a remote environment is an ongoing challenge.

It is fundamental to note that this study has certain limitations. First, this is still in the simulated clinical education phase and has not yet been tested for smart glasses use in a clinical setting. This study shows that smart glasses can be used to demonstrate detailed equipment operation and procedures. In the next phase, it is necessary to study remote demonstration using smart glasses for communication, evaluation, and treatment of physical therapists to patients in a clinical setting. Second, it examined students’ subjective mastery degree but failed to examine objective outcomes such as improved grades and actual learning effect. The effectiveness of learning in practical training using smart glasses should also be investigated. In addition, as noted above, the smart glasses group showed delays in video transmission due to network errors during the demonstration. This may have affected the learning of the smart glasses group. In order to accurately study the effects of learning, it is necessary to take sufficient care with the hardware problems of the smart glasses to conduct the practical program.

In this study, as a feasibility study for remote clinical practice, we first utilized smart glasses for clinical simulation at NMES. Smart glasses would be excellent not only for applying treatment devices, but also for sharing the physical therapist’s point of view and real-time video of the therapist’s hand as they are evaluating and applying treatment techniques. The incorporation of simulated patients into the practice of primary education in physical therapy is recommended in reviews of physical therapy education^31)^. The use of remote devices such as smart glasses could be developed to simulate the clinical setting while communicating with the patient. Further, we would like to continue to examine the possibility of using this system not only for student education, but also for clinical education after graduation.

In conclusion, it was found that sharing visual field information of the teacher using smart glasses provides the mastery of degree comparable to face-to-face demonstrations in physical therapy practice. This is the first report of the use of smart glasses in physical therapy education. On the other hand, issues remained in terms of realism, ease of asking questions, and smooth video sharing. While addressing these issues, it is necessary to conduct research toward an actual remote clinical education system.

## Funding

No funding was received.

## Author contributions

YT and TY led the study, designed the study, performed data measurement and analysis, and wrote the manuscript. YS and YF performed data measurement and analysis. KM, TT, TF, and HD provided advice about data analysis and the manuscript.

## Conflicts of interest statement

The authors declare that there are no conflicts of interest.
